# Ribosome-inactivating proteins in bacteria

**DOI:** 10.1099/mgen.0.001839

**Published:** 2026-09-21

**Authors:** Malak H. El-Ramly, Casey C. Fowler

**Affiliations:** 1 Department of Biological Sciences, University of Alberta, Edmonton, AB, T6G2E9, Canada

**Keywords:** antifungals, exotoxins, ribosome-inactivating proteins, ricin, Shiga toxin, *Spiroplasma*, *Streptomyces*

## Abstract

Ribosome-inactivating proteins (RIPs), a group of biological toxins that depurinate a critical residue within the 28S rRNA, exhibit one of the most potent and elegant mechanisms of cellular toxicity. Examples of RIPs include ricin and Shiga toxin, which are amongst the most notorious toxins produced by plants and bacteria, respectively. Here, we discuss RIPs in the Bacteria domain. We review the biology of Shiga toxin and its role as a transformational virulence factor for some of the most virulent *Escherichia coli* and *Shigella* pathotypes, as well as recent work identifying an assortment of RIP toxins deployed by *Spiroplasma* endosymbionts to protect their host from parasitic attack. We further shed light on the expansive but poorly understood arsenal of proteins with the conserved RIP functional domain that can be found in the genomes of diverse bacterial species, including proteins with complex domain architectures thought to occur only in eukaryotic RIP toxins. Based on the impressive arsenal of RIPs found in *Streptomyces*, as well as the ubiquitous presence of this genus in the niches where many of the other organisms that produce RIPs are found, we speculate that *Actinomycetes* such as *Streptomyces* might have been the evolutionary origin of the RIP domain and hypothesize that *Streptomyces* has played a central role in the spread of RIPs throughout the biosphere.

**Impact Statement**

This article provides a rigorous overview of what is known about ribosome-inactivating proteins (RIPs) in bacteria. This class of toxins has a major impact on human health and also plays important ecological roles. Furthermore, RIPs are commonly utilized agents for the design of novel therapeutics and agricultural technologies. This review not only summarizes what is known about the major classes of bacterial RIPs, but also sheds new light on the spectrum of uncharacterized RIPs that exist in bacteria and the lineages that produce these toxins. This article therefore aims to provide a comprehensive view of RIP biology in bacteria, a resource that is not currently available.

## Data Summary

The authors confirm that all supporting data, code and protocols have been provided within the article or through supplementary data files.

## Introduction

### Ribosome-inactivating proteins

Ribosome-inactivating proteins (RIPs) are a class of biological toxins first discovered in the 1800s [1]. Since then, they have been the subject of great attention due to their impact on human health, as well as their potential as both biological weapons and therapeutics. The ribosome is fundamental to all life on the planet, and many diverse mechanisms have evolved to target ribosomes as a mechanism of toxicity. Here, we limit our discussion to proteins carrying the conserved RIP functional domain, which exhibits RNA *N*-glycosidase activity to depurinate a specific residue of the large subunit rRNA (28S rRNA in eukaryotes). Other proteins can be rightfully considered ‘RIPs’, since their activity also inactivates the function of the ribosome. This includes toxins such as *Burkholderia* lethal factor 1, which deamidates the eukaryotic translation initiator eIF4A [2], and sarcin, which cleaves a specific phosphodiester bond in the same region of the 28S rRNA that is targeted by RIPs [3]. However, these are distinct classes of enzymes from RIPs and are not considered further here.

**Fig. 1.**
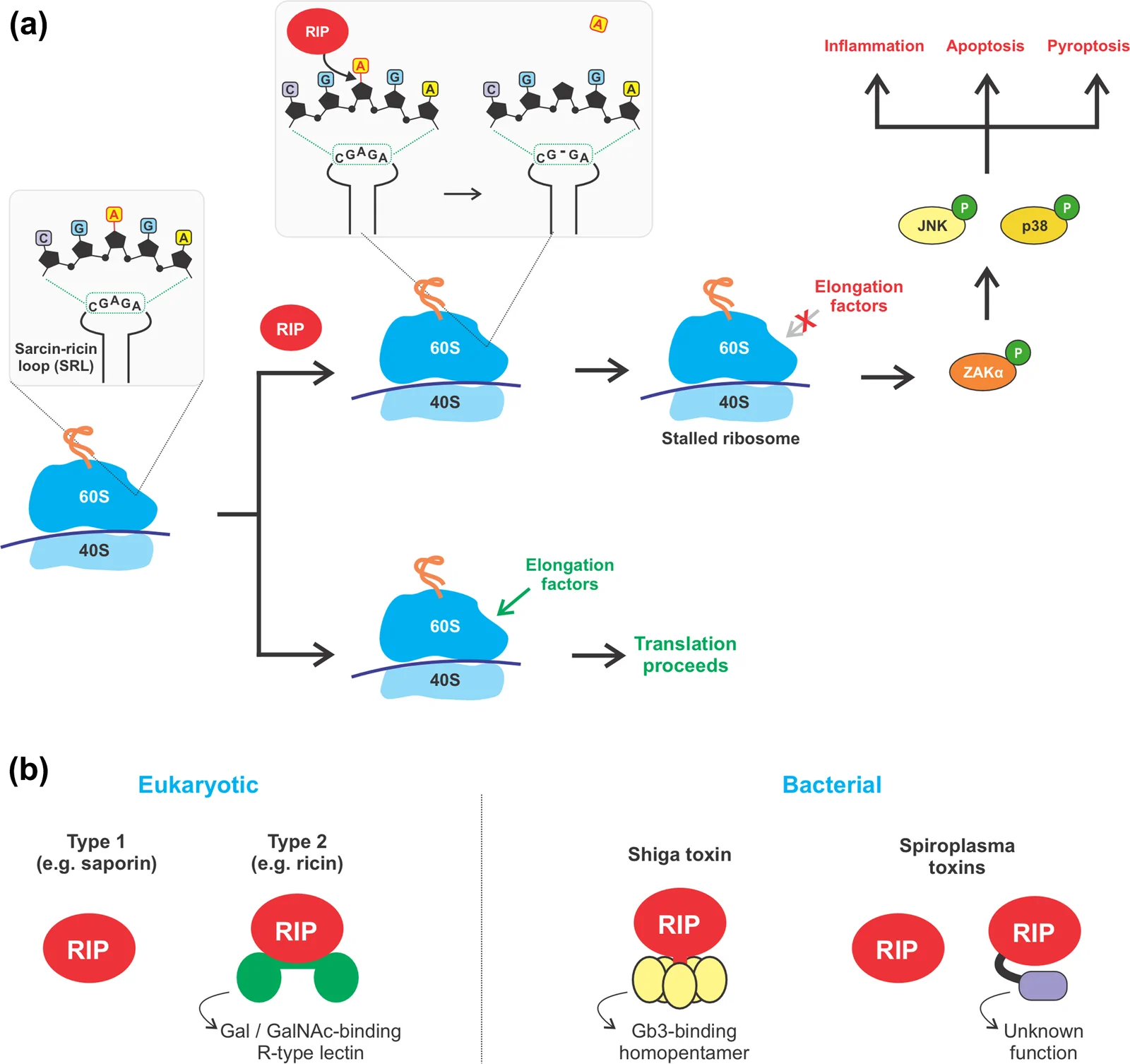
Schematic of RIP architecture, activity and cellular responses. (**a**) Diagram showing the activity of RIPs, which cleave the bond connecting the adenine nucleobase to the ribose for a specific and highly conserved residue within the sarcin–ricin loop (SRL) of the 28S rRNA of eukaryotic ribosomes, setting in motion the ribotoxic stress response. Specifically, depurination of this crucial rRNA residue prevents the recruitment of critical elongation factors, resulting in ribosome stalling, which is sensed by the ribosome-associated MAP3 kinase ZAK*α*. ZAK*α* then autophosphorylates and activates JNK and p38 signalling, which can have a range of downstream effects that depend on the extent and duration of the damage, which can include inflammatory responses and cell death via apoptosis or pyroptosis. (**b**) Schematic showing the architecture of major RIPs, where certain RIPs are composed only of the core RIP domain (e.g. type 1 eukaryotic RIPs and many *Spiroplasma* RIPs), whereas others have additional functional domains or form multi-subunit protein complexes.

RIPs are a class of highly diverse proteins that are widely distributed throughout the biosphere. They are defined on the basis of a common function: they catalyse the cleavage of the bond connecting the adenine base to the ribose for a specific and highly conserved residue of the 28S rRNA, a major component of the large subunit of eukaryotic ribosomes (Fig. 1). The depurination of this residue, A4324 in rat liver rRNA, potently inactivates ribosomal function due to the essential role that this region, known as the sarcin–ricin loop, plays in recruiting translation elongation factors required for protein synthesis [4–8]. At sufficiently high concentrations, certain RIPs have been observed to depurinate other nucleic acids, including DNA and poly(A) tails, although the importance of this, if any, is not clear [9, 10]. Similarly, the relevance of the finding that certain RIPs can depurinate bacterial ribosomes is unclear [11, 12], and we are unaware of any RIP toxins that possess delivery mechanisms to efficiently overcome the unique challenge of crossing bacterial cell envelopes. However, given the diversity of RIPs and the evolutionary advantages of deploying antibacterial mechanisms, it would not be surprising if RIP toxins that target bacteria exist.

Although highly divergent RIPs are scattered throughout the biosphere, it is likely that they all share a common (and ancient) ancestor. Several factors strongly argue against the possibility that RIPs independently evolved in different branches of life. This includes pronounced similarities in structure amongst all characterized RIPs, the conservation of amino acid (aa) residues that play key structural and functional roles and the highly specialized activity and mechanism of RIPs. For example, the experimentally determined structures of the highly divergent RIPs ricin and Shiga toxin show strong structural conservation including remarkably similar active sites that feature conserved residues in nearly identical positions [13, 14]. Active site residues broadly conserved in RIPs include a glutamate and an arginine (E167 and R170 in Shiga toxin StxA) that are directly involved in catalysis, a pair of tyrosines (Y77 and Y114) that coordinate the purine ring of the substrate adenine, and a conserved tryptophan (W203) proposed to play a role in maintaining the conformation of the active site [13, 15–18].

Given the central role the ribosome plays in cellular function, it is not surprising that RIP activity elicits severe and complex effects in intoxicated cells. A central pathway that dictates the cellular response to RIP activity is the ribotoxic stress response (RSR) pathway (Fig. 1a) [19]. The RSR is initiated by a MAP3 kinase known as ZAK*α*, which interacts with ribosomes and senses the ribosome stalling that occurs in response to RIP activity [20, 21]. This triggers ZAK*α* autophosphorylation and its subsequent activation of p38 and JNK signalling pathways. The ultimate outcomes of the RSR pathway can be variable and complex and can depend on the magnitude and the duration of the ribosomal damage [20, 21]. For potent RIPs such as ricin or Shiga toxin, even a low number of RIP molecules within a cell can be sufficient to cause complete or near-complete inactivation of ribosomal activity, and the strong RSR that ensues culminates in cell death [20, 22]. Correspondingly, low doses of RIPs can be sufficient to cause substantial tissue damage, leading to the very potent toxicity that is observed for some RIPs [23, 24]. However, although the mechanism of RIPs is well conserved, they can exhibit drastically different levels of toxicity. Most critically, RIPs have intracellular targets and thus require mechanisms for cellular uptake and trafficking in order to access ribosomes and elicit damage. RIPs can exhibit drastic differences in this regard, which can be a predominant factor underlying the level of toxicity of a particular RIP toxin toward a particular organism. Furthermore, different RIPs have evolved to target the ribosomes of different species and thus can show marked variation in their capacity to depurinate the ribosomes of a particular species [25, 26]. The sarcin–ricin loop is highly conserved, but a major factor underlying the different activity levels can be the nature of the RIPs’ interactions with ribosomal proteins in the context of the complete ribosome structure [25, 27].

### RIPs in eukaryotes

The majority of the RIPs that have been studied to date are produced by eukaryotic species, predominantly plants. Eukaryotic RIPs are often divided into categories on the basis of their domain architecture. Type 1 RIPs consist of a single enzymatic RIP domain, whereas type 2 RIPs also include associated lectin domains that interact with glycans on the surface of target cells to mediate cellular uptake (Fig. 1b). Type 3 RIPs, which are comparatively rare, exhibit domain architectures that differ from the two canonical RIP types. RIPs are broadly distributed, although far from ubiquitous, in flowering plants and are produced by both monocots and dicots [28]. The best-studied eukaryotic RIP is ricin, which is produced by the castor plant, *Ricinus communis* [29]. Ricin is a type 2 RIP and is potently toxic to animals, including humans [24]. Its potency, coupled with a relative ease of access, has led to concerns over its use as a bioterrorism agent; it is considered by the US Centers for Disease Control and Prevention (CDC) as a Category B bioterrorism agent. Ricin, like other type 2 RIPs, does not exhibit strong specificity for a particular cellular receptor, but instead, its lectin recognizes a range of glycans terminating in galactose or GalNac, found on both glycolipids and glycoproteins [30]. Following binding to these cell surface markers, ricin is internalized by endocytosis, trafficked in a retrograde fashion to the endoplasmic reticulum (ER), after which the RIP domain is translocated across the ER membrane into the cytoplasm, where it can access target ribosomes [31]. By contrast, type 1 RIPs, which often lack a mechanism of cellular uptake, are generally less toxic and have been isolated from commonly consumed plants such as pumpkins and tomatoes [32, 33]. However, the situation is not always this simple. For example, certain type 2 RIPs exhibit very low levels of toxicity, such as cinnamomin, which is produced by the seeds of camphor trees [34]. This is believed to be due to its lectin domain, at least in part, since the cytotoxicity of the cinnamomin A chain (RIP) is substantially increased when fused to the ricin B chain (lectin) as a chimeric protein [35, 36].

The function of plant RIPs, by which we refer to how they benefit the plant that produces them, is not always clear. It is likely that different functions have evolved. One likely explanation for type 2 RIPs, which are potently toxic to animals, is that they serve as a defence mechanism. For example, ricin is predominantly produced in seeds, and this could serve as a deterrent to prevent herbivores or insects from consuming *R. communis* seeds [37]. RIPs have also been implicated in defence from viral and fungal infections [38], potentially by targeting and shutting down the plant’s own ribosomes as a means to deter the spread of the infection to other cells [39, 40]. It has also been proposed that certain RIPs might be involved in regulating senescence in plants [41].

Beyond plants, RIPs are generally rare in Eukarya, but RIPs are produced by certain fungi. A noteworthy example of this is mucoricin, a recently identified type 2 RIP that has been implicated as a virulence factor in mucormycosis, an opportunistic fungal infection by species of the Mucorales order [42]. Interestingly, although this toxin was shown to exhibit RIP activity, it is substantially smaller than characterized RIPs such as ricin, and it exhibited reduced RIP activity [42]. Type 1 RIPs have also been found in metazoan genomes, including insects such as mosquito and related whitefly species; the function and activity of these proteins are not understood [43–45].

### Aims and scope of this review

In this article, we summarize what is known about RIPs in the Bacteria domain. This includes reviews of Shiga toxin, a potent bacterial virulence factor that has a substantial impact on human health, as well as RIP toxins produced by *Spiroplasma* arthropod endosymbionts that are proposed to protect their host from parasites. Table 1 summarizes key information for these two classes of bacterial RIPs. Although these two families are the only well-characterized bacterial RIPs, genome sequencing data indicates that they are only the tip of the iceberg, and a tremendous wealth of bacterial RIPs await characterization. We shed further light on the spectrum of bacterial lineages where putative RIPs can be found and highlight the sequence and architectural diversity of uncharacterized bacterial RIPs. A noteworthy outcome of this analysis is the appreciation that *Streptomyces* encode a large and diverse repertoire of RIPs. We discuss the implications of this finding for hypotheses on the origins of RIPs and how they have spread throughout the biosphere.

**Table 1.** Summary of key information for the two major classes of characterized bacterial RIPs

	Shiga toxin	*Spiroplasma* RIP
**Produced by**	Shiga toxin-producing *E. coli* (STEC), *Shigella dysenteriae* type 1, rare strains of misc. other species	*Spiroplasma* endosymbionts of flies
**Toxin diversity**	Two major types, Stx1 and Stx2; structurally and functionally similar; important activity differences between variants	Substantial strain-to-strain differences, with as many as 8 RIPs in a given strain; much diversity in RIPs, with important functional differences between variants
**Toxin locus/spread**	Encoded within prophages; toxin expression/secretion associated with phage induction	Encoded within chromosome or on plasmids; genome duplication, horizontal transfer; conjugative plasmid-mediated transfer?
**Toxin architecture**	AB5-type toxin; RIP (StxA) subunit forms toxin complex with 5 glycan-binding delivery subunits (StxB)	Unknown, but appears to lack a delivery subunit, similar to eukaryotic type 1 RIPs
**Cell targeting/entry**	StxB binding Gb3, a glycolipid receptor on the surface of target cells, leading to endocytic uptake	Unknown; role of accessory domains for some RIPs? Pore-forming toxin encoded adjacent to RIPs in rare cases?
**Target of RIP**	Host species during infection	Parasitic organisms (nematodes, wasps) that prey on host
**Function**	Unknown; interference with host immune function? Nutrient acquisition? Defence against predatory eukaryotic microbes?	Protect their host from parasites, thereby promoting host survival. This provides a fitness advantage that favours their carriage
**Impact**	Cause of haemorrhagic colitis and haemolytic uraemic syndrome associated with infections; major impact on human health	Potentially an important ecological impact through protecting host from predation

For those who are interested, we point readers to helpful reviews focused on eukaryotic RIPs [1, 46], or on the applications of RIPs [1, 47]; these topics are not discussed extensively here.

### Shiga toxin

#### Shiga toxin structure: a unique AB_5_-type toxin

The most infamous and best-studied bacterial RIPs are Shiga toxins, a small family of closely related toxins that are key virulence factors for pathogenic *Shigella* and *Escherichia coli*. Although bacterial RIPs are rarely discussed in terms of the ‘types’ devised for eukaryotic RIPs, Shiga toxin is best thought of as a type 2 RIP, since its RIP subunit forms a complex with glycan-binding subunits that mediate uptake into target cells. Shiga toxin is an AB_5_-type toxin, meaning that it is composed of a single active (A) subunit, which mediates the toxin’s effects, in complex with five binding (B) subunits, also known as delivery subunits, which are receptor binding subunits that mediate toxin uptake and trafficking in target cells (Fig. 1b) [48, 49]. The Shiga toxin delivery platform is a homopentamer of StxB, a ~70 aa B subunit, which assembles with the A subunit, StxA, a ~300 aa RIP, to produce the holotoxin [13]. In addition to Shiga toxin, other toxins from the AB_5_ family include cholera toxin, pertussis toxin and typhoid toxin, each of which is a principal virulence factor for a major human pathogen [50–52]. AB_5_-type toxins therefore have an oversized impact on human health and can be promising targets for therapeutic approaches to combat bacterial diseases. All known AB_5_-type toxins adopt a similar global architecture, wherein the B subunit pentamer forms a donut-like ring structure at the base of the toxin, and the A subunit is perched on top [48, 49]. The toxin complex is held together in large part by the insertion of an *α*-helix from the A subunit into the central pore of the B subunit pentamer.

Despite this common architecture, the individual subunits of different AB_5_ toxins often share little or no significant similarity in sequence, structure or function [48, 53]. Shiga toxin, for example, is the only known AB_5_ toxin that utilizes a RIP as an active subunit, and its B subunit differs substantially in sequence, structure and receptor binding compared to those from other AB_5_ toxins. StxA is composed of two domains, A1 and A2, where A1 is the RIP domain and A2 is a small domain that mediates interaction with the B subunits [13, 54, 55]. As noted above, StxA adopts a structure that has significant similarities to other RIPs, including a conserved active site. These similarities, however, are limited to the A1 domain, and the C-terminal A2 domain is unique to Shiga toxin [13]. In terms of the delivery platform, in addition to adopting the common pentameric ring architecture found in all AB_5_ toxins, the StxB core also features the oligonucleotide/oligosaccharide-binding fold, a common structural motif that facilitates ligand binding found in other AB_5_ toxin B subunits, as well as many other proteins [53]. Despite these similarities, StxB is smaller and is structurally distinct from other AB_5_ B subunits, and its mechanisms of glycan binding are distinct as well. The StxB homopentamer has 15 total glycan-binding sites, three per monomer, with some being formed at the interface between adjacent monomers [56]. If we consider StxA to sit on top of the StxB homopentamer, the glycan-binding sites would all reside on the bottom, which represents the receptor-binding face of the toxin.

### Shiga toxin distribution

Shiga toxins are predominantly found in the *Shigella*/*E. coli* group. *Shigella* is commonly described as a distinct genus for historical reasons, as well as reasons that relate to its distinct pathogenesis and its medical importance. However, from a phylogenetic perspective, *Shigella* fall within *E. coli*, a very large and diverse bacterial species [57, 58]. Shiga toxin is perhaps best thought of as a single toxin that has a number of sequence variants. There are two primary groups of Shiga toxins, Stx1 and Stx2, which are structurally and functionally similar, but which share only ~60% sequence identity [54, 59]. Both major types can be further divided into subtypes (e.g. Stx1a), with a larger number of sequence variants having been identified for Stx2. In *Shigella*, Shiga toxin production was originally considered a characteristic of *Shigella dysenteriae* serotype 1, but it has since been observed that Shiga toxin is produced by scattered strains of other Shigellae, including both different *S. dysenteriae* serotypes as well as other *Shigella* species [60–62]. In *E. coli*, strains that encode the Shiga toxin genes are collectively referred to as Shiga toxin-producing *E. coli* (STEC) and are a defining feature of the *E. coli* pathotype Enterohaemorrhagic *E. coli* (EHEC) [63]. Over 100 *E. coli* serotypes are known to encode Shiga toxin, but the majority of human cases of EHEC are caused by a handful of more common serotypes, most prominently O157:H7 [64, 65]. There are scattered reports of bacterial strains encoding Shiga-like toxins beyond the *Escherichia*/*Shigella* group [66–69]. The relevance of this for human health is generally unknown, but clinical reports of enhanced virulence, such as a documented case of Shiga toxin–encoding *Enterobacter cloacae* infection causing haemolytic uraemic syndrome (HUS), suggest that these horizontally acquired Shiga toxins can impact pathogenesis [66]. Given the scarcity of such strains, however, it is likely that they impact a limited number of patients.

### Shiga toxin’s biological programme

Shiga toxins are prophage-encoded toxins, and both Stx1 and Stx2 are generally carried on lambdoid bacteriophages [70, 71]. Shiga toxin’s biological programme is integrated into that of its phage host; the *stxA* and *stxB* genes are located upstream of the phage’s lysis cassette, and their expression is driven by phage promoters. Their expression is induced alongside late phage genes by conditions that trigger phage activation and phage-mediated lysis, such as DNA damage [72, 73]. Following DNA damage, the bacterial SOS response is induced, leading to RecA-mediated cleavage of CI, the phage transcriptional repressor, which promotes the expression of the *stx* genes and of the late phage genes [74]. Upon toxin expression, it is translocated to the periplasm where it assembles. The holotoxin is subsequently released into the intestinal lumen by phage-induced cell lysis [72, 73]. Because antibiotics can induce bacterial cell lysis, and certain antibiotic classes can also stimulate prophage induction (and thus Shiga toxin production), the use of antibiotics to clear infections by Shiga toxin-producing bacteria is generally not recommended, since it can expose the patient to higher levels of Shiga toxin and thus increase the risk of serious complications [75–77].

Following Shiga toxin’s release into the intestinal lumen, the StxB homopentamer specifically recognizes the glycosphingolipid globotriaosylceramide (Gb3) as a receptor to mediate host cell binding and uptake. Avidity, the binding of multiple Gb3 molecules by the multitude of binding sites on the StxB pentamer, is thought to be an important feature of receptor binding [78–81]. Binding triggers Shiga toxin endocytosis into target cells, which has been observed to occur via both clathrin-dependent and clathrin-independent mechanisms [82–84]. The toxin then traffics from early endosomes in a retrograde fashion via the trans-Golgi network to the endoplasmic reticulum [85]. During trafficking, a linkage between the A1 and A2 domains of StxA is cleaved by membrane-bound furin proteases [55, 86]. It has also been proposed that this protease-sensitive A1–A2 linkage can be cleaved by proteases within the intestinal lumen, and it is possible that both mechanisms are relevant *in vivo* [87]. Upon cleavage, the A1 and A2 domains initially remain associated in the holotoxin complex due to an intramolecular disulfide bond between cysteines from either domain. This bond is reduced within the ER lumen, freeing the A1 RIP catalytic domain, which exploits the ER-associated protein degradation pathway to translocate across the ER membrane and into the cytoplasm [85, 86, 88]. Once it reaches the cytoplasm, the StxA A1 domain refolds and binds to the ribosomal P-stalk, similar to many other RIPs, including ricin [27, 89, 90]. This binding orients the active site to the sarcin–ricin loop, leading to depurination of the crucial 28S rRNA residue and the initiation of the RSR as outlined in the Introduction and shown schematically in Fig. 1.

The cellular response to Shiga toxin intoxication is variable and depends on factors such as cell/tissue type, toxin concentration and the duration of exposure [85, 91]. Shiga toxin’s stimulation of the RSR activates key MAPK pathways including the JNK, p38 and ERK pathways, and the resulting signalling cascades can culminate in cell death. Apoptotic cell death has been commonly reported; however, a recent study suggests that pyroptosis is a more relevant outcome than was previously appreciated [85, 92, 93]. Beyond its direct effects within intoxicated cells, Shiga toxin can also induce local inflammation since intoxication can stimulate the production of pro-inflammatory cytokines [85, 91]. It is proposed that the release of cytokines such as tumour necrosis factor-alpha and IL-1*β* can induce the upregulation of Gb3 in local cells, promoting increased toxin uptake and exacerbating disease [94–96].

### Shiga toxin and disease

Shiga toxin is a transformational virulence factor that is directly responsible for the most severe disease outcomes associated with the *Shigella* and EHEC that produce this toxin. Both *Shigella* and EHEC are food-borne pathogens that are transmitted primarily through the ingestion of contaminated food and water. While humans are the only known reservoir for *Shigella*, livestock, cattle in particular, are known to be major reservoirs of EHEC [97]. Infections are often characterized by fever, nausea and bloody diarrhoea and, in more severe cases, haemorrhagic colitis which can progress to HUS. HUS, which occurs in ~5–15% of EHEC cases, is a life-threatening disease that stems from kidney damage [98–100]. *S. dysenteriae* type 1 has historically been the cause of major pandemics, but its contemporary global burden is relatively low [58]. EHEC remains an important pathogen worldwide, with previous estimates of the global burden topping 2 million cases per year [101]. Amongst the commonly encountered Shiga toxin subtypes, Stx2 is generally more potent in experimental infection models, which is consistent with the observation that Stx2-encoding strains are more commonly associated with severe clinical outcomes [23, 102–104]. Interestingly, while most Shiga toxin subtypes specifically bind Gb3, Stx2e strongly binds globotriaosylceramide (Gb4) [105]. Strains encoding Stx2e are not generally human pathogens, but are known to cause outbreaks leading to oedema disease in pigs, which produce elevated levels of Gb4 [105, 106].

Shiga toxin is not the only virulence factor for the pathogens that produce it. For example, EHEC also generally carry the locus of enterocyte effacement (LEE) pathogenicity island, which encodes a type III secretion system and associated effector proteins [63]. The LEE pathogenicity island is particularly crucial for colonization due to its role in adherence to enterocytes [107]. However, EHEC strains have been identified that lack LEE and which present similarly in the clinic [108, 109]. Furthermore, *E. coli* strains similar to EHEC that do not encode Shiga toxins do not cause the haemorrhagic colitis and HUS that define the EHEC pathotype [110]. This is in strong support of the dogma that Shiga toxin is the direct cause of these symptoms. The intestinal epithelium is generally thought to produce low levels of Gb3 and is resistant to Shiga toxin. Shiga toxin’s most serious effects stem from its activity once the toxin migrates beyond the intestines, spreading via the blood to the kidneys, or potentially other sites. How Shiga toxin accesses the bloodstream is not clear; transmigration of the toxin across an intact epithelial layer has been proposed and shown in experimental systems [111, 112]. However, it is also possible that, despite generally low levels of Gb3 in the intestinal epithelium, toxin-induced damage that disrupts the intestinal barrier is also a significant factor. Once Shiga toxin spreads systemically, it disproportionately impacts the kidneys, in large part due to the high levels of Gb3 expression in certain renal cell types, most notably podocytes and the endothelium [113–115]. HUS is a direct result of the damage elicited by Shiga toxin to the kidneys, both through direct tissue damage and via inflammatory responses to intoxication [91, 100]. Although rare, systemic spread of Shiga toxin can also have serious neurological impacts [116].

### *Spiroplasma* RIP toxins

Beyond Shiga toxins, the other bacterial RIPs that have been well characterized are those encoded by *Spiroplasma*, a genus of small parasitic bacteria that lack a cell wall. *Spiroplasma* are widespread and diverse, but are commonly endosymbionts of arthropods including *Drosophila. Drosophila* is a genus of small flies that includes the fruit fly *Drosophila melanogaster*, which has served as an important model organism in genetics [117, 118]. *Spiroplasma* uses a trans-ovarian mechanism of transmission in *Drosophila* and persists extracellularly within host haemolymph [119, 120]. Because they can impose a fitness cost on their host, and maternal transmission is not infallible, *Spiroplasma* require mechanisms to ensure they are maintained by their host. Bacterial endosymbionts of arthropods have developed various strategies to ensure their retention, which includes the production of toxins that manipulate the physiology of their host or that target their host’s predators as a means to promote host survival [121, 122]. Over the last decade, compelling evidence has been compiled supporting the hypothesis that RIP toxins produced by *Spiroplasma* endosymbionts of *Drosophila* species play a key role in protecting its host from predatory parasites including nematodes and wasps.

The role of *Spiroplasma* RIPs was first characterized in a *Spiroplasma* symbiont (dubbed *s*Neo) of *Drosophila neotestacea*, in the context of parasite defence against *Howardula* nematodes [123]. *Howardula* infect the haemolymph of the flies, and shed juveniles within the haemocoel, which ultimately sterilizes *Drosophila* and often kills them. The presence of *s*Neo, however, confers a substantial protective effect for the host against the parasitic nematode [117, 123, 124]. Following up on transcriptomic analyses that indicated that a putative *s*Neo RIP was strongly induced in response to nematode parasitization, Hamilton *et al*. purified this protein and demonstrated that it is a *bona fide* RIP that is able to depurinate *Howardula* ribosomes *in vitro* [123, 125]. Further experiments demonstrated that the ribosomal damage elicited by RIPs to the nematode is also observed *in vivo*, when the flies were co-infected with the symbiont and parasite. An ensuing study demonstrated that this effect can be observed beyond this specific model system [126]. In an infection model, it was shown that *Spiroplasma* endosymbionts of both *D*. *neotestacea* (*s*Neo) and *D*. *melanogaster* (*s*Mel) elicit high levels of RIP activity against the ribosomes of parasitoid wasps. This RIP activity, which was observable shortly after the hatching of the invading wasps, is proposed to be an important factor driving the high levels of wasp mortality conferred by *Spiroplasma* in these experiments. Notably, much lower levels of *Spiroplasma*-induced ribosomal depurination (and no significant drop in the level of intact ribosomes) were observed for the wasp *Pachycrepoideus vindemmiae*, which correlates with the observation that this wasp is resistant to *Spiroplasma’s* defence mechanisms [126]. Similarly, *s*Mel does not exhibit detectable RIP activity against parasitic nematodes during infection, and it also fails to protect its host against nematode parasitization [127]. Technical limitations preclude more direct assessments of the role of *Spiroplasma* RIPs in host defence, such as generating mutant strains that lack RIPs and assessing the impact of these mutations on *Spiroplasma*’s capacity to protect *Drosophila* from infection. However, the high levels of ribosome depurination observed for parasites during infection and the correlation of *Spiroplasma*’s RIP activity with its capacity to protect the host imply that this may be an important aspect of how *Spiroplasma* protects *Drosophila* from parasites and, in turn, the capacity of *Spiroplasma* to promote its carriage within the host.

There is a great deal of diversity in terms of the number and nature of RIPs encoded by different *Spiroplasma* endosymbionts that suggests that different RIPs might be deployed to target different species or in different contexts. The RIPs exist in multi-copy gene families, with some strains encoding up to eight different RIP genes [26, 121, 127]. A recent study that examined the activity of an assortment of purified *Spiroplasma* RIPs demonstrated that each was active *in vitro* against purified ribosomes from various eukaryotic species, confirming that they are *bona fide* RIPs [26]. The activity of the RIPs varied when assayed against ribosomes from different species, and the RIPs also varied in their ability to be taken up by live cells. Similar to type eukaryotic 1 RIPs, *Spiroplasma* RIPs lack an overt delivery subunit or mechanism, and it is currently unclear how they enter cells in order to elicit their activity. Certain (rare) RIP genes are encoded adjacent to a potential pore-forming toxin with homology to epsilon toxin in *Clostridium* [127], which could potentially represent one mechanism by which the RIPs access parasite ribosomes. It is also noteworthy that some *Spiroplasma* RIPs have accessory domains of unknown function (Fig. 1b). These domains could potentially mediate cell uptake, although this has not yet been demonstrated [26, 123, 127]. In fact, in some instances, the variable accessory domains appear to have an inhibitory effect on RIP activity under certain test conditions [26]. One possible explanation for this could be that the function of these accessory domains is to inhibit RIP activity against the host (*Drosophila*) as a means of protecting the host organism against collateral damage. This could help explain the substantially greater RIP intoxication of parasites compared to hosts observed in infection models [123, 126].

### The distribution of bacterial RIP proteins

Beyond Shiga toxin and the RIPs produced by *Spiroplasma*, the biology and ecology of RIPs in bacteria are not well understood. Previous work has shown that putative RIPs are encoded in diverse bacterial lineages [128, 129]. In many cases, these instances appear to represent horizontal acquisitions by rare strains of lineages that generally lack RIPs. Scattered uncharacterized RIPs have been identified in various genera such as *Rickettsiella*, *Flavobacterium* and *Burkholderia* [128, 129]. This patchy distribution is consistent with their predicted function in mediating interactions with hosts or competing microbes. Factors such as this can represent a ‘double-edged sword’; they can confer a substantial competitive advantage, but can also potentially disrupt the local community/environment in a way that is ultimately detrimental. Deploying a potently toxic molecule like a RIP also selects for resistance mechanisms. So, while RIP proteins can provide a substantial benefit to a bacterium, this requires a suitable delivery mechanism to target and access intended cell(s), as well as a specific context in order to be beneficial.

The RIP domain is well characterized and readily recognized by protein domain analysis tools. As a means to examine the diversity and distribution of RIPs in bacteria, we queried the NCBI Conserved Domain Database for bacterial proteins with the RIP domain [130]. This analysis revealed a diverse list of bacterial lineages that encode putative RIP domain-containing proteins. Although some of these entries might not represent *bona fide* RIPs, and some deposited sequences might not cite the correct source species, the spectrum of species identified as encoding RIPs strongly supports the notion that this family of proteins is very broadly distributed in Bacteria. Examples of lineages with multiple putative RIP protein entries include the prominent fish pathogen *Aeromonas salmonicida* [131]; *Haematospirillum jordaniae*, a poorly understood Gram-negative Alphaproteobacterial species that has emerged as a rare human pathogen capable of causing severe disease [132]; and various *Microbulbifer* species, which are common Gammaproteobacterial members of marine microbiomes [133]. Despite the strong bias of sequence databases for bacterial pathogens such as STEC, the bacterial lineage harbouring the largest number of sequenced RIP proteins was *Streptomyces*, which represented more than 1,900 of the <3,500 bacterial entries. Related *Actinomycetes*, such as *Kitasatospora* and *Micromonospora*, accounted for an additional ~300 entries. Indeed, although the *E. coli/Shigella* group is where RIP toxins are most notorious in bacteria, RIPs are narrowly distributed and exhibit very limited sequence diversity in this large and diverse taxon. By contrast, putative RIPs are common in *Streptomyces* and are found in a myriad of different *Streptomyces* species. In the following section, we summarize what is known about RIP toxins in *Streptomyces* and shed light on the tremendous diversity of uncharacterized putative RIP proteins that can be found in *Streptomyces* genomes.

### *Streptomyces* encodes a rich diversity of RIP proteins

*Streptomyces* is a genus of spore-forming Gram-positive bacteria renowned for their complex life cycle and as producers of a staggering diversity of natural products. These include many of the antibiotics currently used in medicine and agriculture, as well as other molecules that have found clinical utility [134, 135]. *Streptomyces* are ubiquitous and highly abundant in soil and are found in a variety of aquatic environments. They can also be found as symbionts of organisms ranging from plants to insects to marine invertebrates [136, 137]. Certain *Streptomyces* are phytopathogens, but this is the exception rather than the rule, and *Streptomyces* are considered beneficial microbes in most cases [136, 137]. *Streptomyces* produce a large and diverse assortment of antimicrobial compounds in a tightly regulated manner [135]. Although various applications of this antimicrobial repertoire have presumably evolved, this is generally thought to be a strategy to protect local resources at crucial stages of their life cycle. Bacteria are the targets of many such molecules, but *Streptomyces*’ antimicrobial arsenal contains diverse antifungal molecules as well, which is in line with the fact that fungi are prominent members of the soil communities inhabited by many *Streptomyces* [135, 138]. It is therefore not surprising that RIPs are common in *Streptomyces*, a lineage that specializes in producing diverse molecules that are potently toxic to local competitors.

**Fig. 2.**
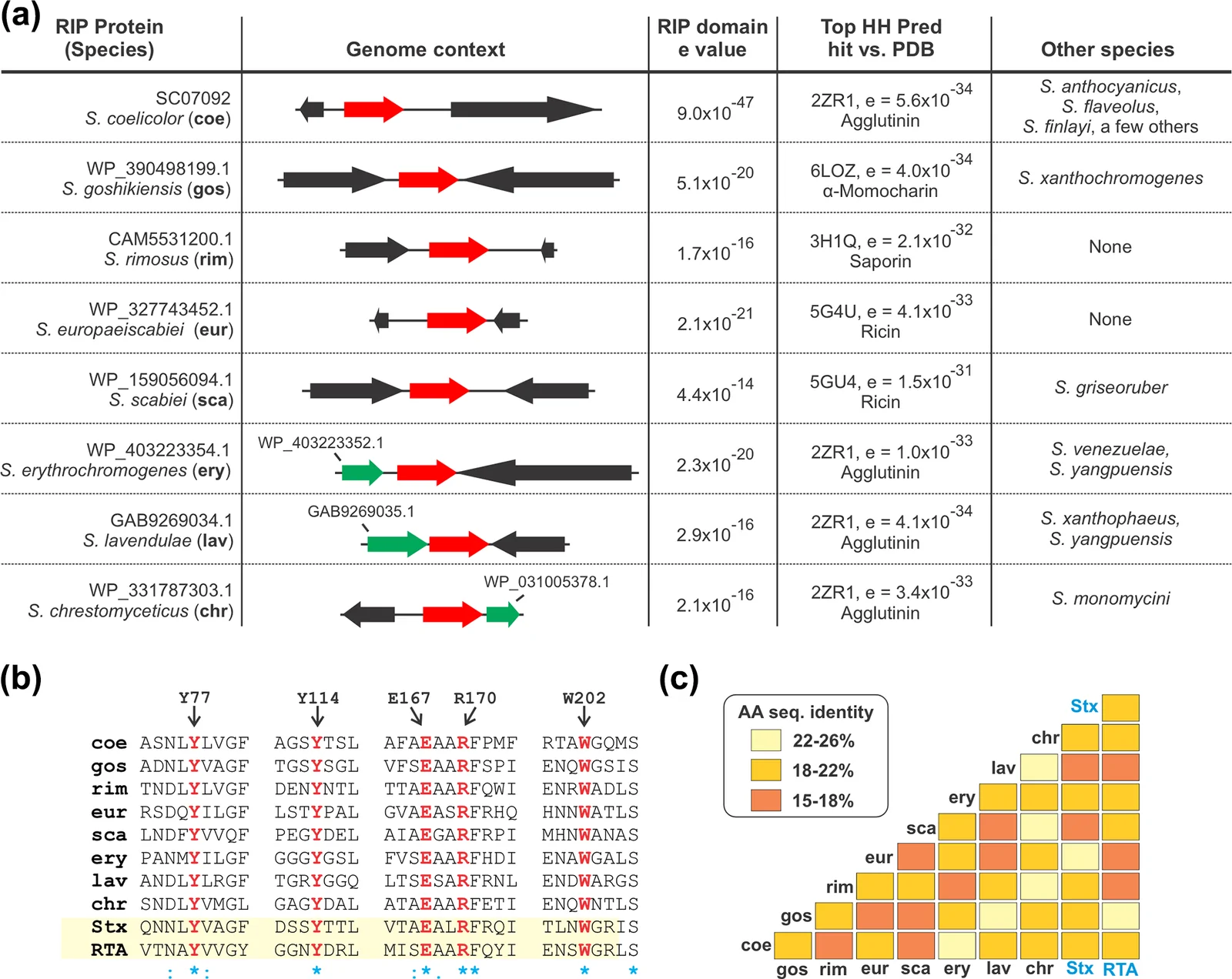
*Streptomyces* encode a diverse repertoire of RIPs. (**a**) Eight representative RIP proteins were arbitrarily selected from the list of *Streptomyces* proteins with putative RIP function domains in the NCBI Conserved Domain Database. The selections were arbitrary, with the goal of selecting high-confidence RIP proteins with diverse sequences from assorted species. The panel provides an overview of key features of each protein, including its NCBI accession numbers, its genomic context, e values describing the confidence for the RIP domain prediction via the NCBI CD Search tool, the top hit for each RIP when queried by HHpred against the PDB and the list of other *Streptomyces* species that encode a protein that is >80% identical over >90% of the query sequence. For genomic context, the red arrow represents the RIP, black arrows indicate neighbouring genes and green arrows represent adjacent genes that could potentially represent delivery mechanisms. Potential delivery genes were identified using HHpred searches for all neighbouring genes – those with significant similarity to proteins with functions such as glycan binding or cell envelope perturbation were flagged; further details are provided in the main text. (**b**) Sequence alignments were performed using muscle (via https://www.ebi.ac.uk/) for the eight *Streptomyces* RIPs from (**a**) as well as the characterized RIPs from Shiga toxin (StxA) and ricin (RTA). Select segments of this alignment are shown that contain conserved residues from the RIP active site (red, numbered according to their aa number in StxA). (**c**) Pairwise percent identity matrix for the sequence alignment described for (**b**). Each combination of RIPs exhibited between 15 and 26% sequence identity, which is illustrated according to the legend.

It was previously noted that *Streptomyces* RIPs can have highly divergent sequences [128]. To expand on this observation, we explored the *Streptomyces* RIP protein entries in the NCBI Protein Domain Database (from the search described above) and found a remarkable amount of sequence diversity. Indeed, when a small set of *Streptomyces* RIPs was randomly sampled, it was generally observed that most or all of these proteins share less than 50% sequence identity with each of the others. To illustrate this diversity, we compiled a list of eight putative RIPs encoded by different *Streptomyces* species (Fig. 2). This set came from sampling only a small fraction of the available entries and likely represents the ‘tip of the iceberg’ with respect to the diversity that exists. Although experimental validation would be required to confirm their RIP activity, each of these eight representative proteins has clear hallmarks of being a *bona fide* RIP: (i) each is identified as having a RIP domain by the NCBI CD-Search tool with very high confidence (e value 1×10^−10^ or less) (Fig. 2a), (ii) querying these proteins against the Protein Data Bank (PDB) of structurally characterized proteins using the HHpred HMM homology search tool revealed hits to characterized RIPs with e values <10^−30^ (Fig. 2a) [139] and (iii) a multiple sequence alignment of the eight representative RIPs, as well RIP domains from Shiga toxin (StxA) and ricin (RTA), revealed that five critical active site residues that are broadly conserved amongst characterized RIPs are also conserved amongst all eight representative *Streptomyces* RIPs (Fig. 2b). Remarkably, RTA, StxA and the eight representative *Streptomyces* RIPs exhibit only ~16–26% aa identity with one another. This analysis indicates that *Streptomyces* encodes large numbers of RIPs that are as different from one another as ricin is from Shiga toxin.

To our knowledge, the only *Streptomyces* RIP that has been characterized to date is SC07092 from the lab strain *Streptomyces coelicolor* A3(2) [140]. Based on *in vitro* translation assays and an RT-qPCR assay to probe for the defining rRNA depurination activity, this protein was shown to be a *bona fide* RIP. Heterologous expression of this protein in the model yeast species *Saccharomyces cerevisiae* indicated that it is potently toxic; however, it had no effect on the viability of a small panel of fungi when applied exogenously, indicating that it is unable to enter the cell and/or access the cytosol of the tested species [140]. The delivery mechanism of the *Streptomyces* RIPs therefore remains unknown. SC07092, as well as each of the other seven *Streptomyces* RIPs we selected for analysis, have high-confidence N-terminal secretion signals according to sequence analysis by SignalP 5.0 [141], strongly suggesting that they do not exert their function within the producing cell, but rather are secreted to intoxicate neighbouring cells. Interestingly, based on HHpred analysis of neighbouring genes, three of the eight representative *Streptomyces* RIPs are encoded in an apparent operon with a gene that could potentially represent a delivery subunit (Fig. 2a, green arrows). This includes the putative RIP from *Streptomyces erythrochromogenes*, which is encoded downstream of a gene that is a strong hit (e value 1.9×10^−13^) to the UDP-Glc binding domain of GalE from *Thermotoga maritima*, and the putative RIP from *Streptomyces lavendulae*, which is encoded downstream of a gene that is a strong hit (e value 6.9×10^−18^) to ostreolysin A from *Pleurotus ostreatus*, a toxin that binds membrane lipids and forms a pore. Most intriguingly, however, is the putative RIP from *Streptomyces chrestomyceticus*, which is encoded downstream of a gene annotated as a ‘RICIN domain containing protein’ and which is a strong hit by HHpred analysis (e value 1.9×10^−13^) to the CdtC cytolethal distending toxin delivery subunit from *Actinobacillus actinomycetemcomitans*. Upon solving the structure of cytolethal distending toxin, it was noted that its CdtA/CdtC delivery subunits exhibit substantial structural similarity to ricin’s lectin domains and that their global arrangement with respect to the A subunit also resembled that of ricin [142]. This analysis suggests that, at least in certain cases, *Streptomyces* RIPs may behave as AB-type toxins and enter target cells via the activity of an adjacent gene that serves as a delivery subunit.

Many *Streptomyces* RIPs are not encoded adjacent to a potential delivery subunit. It is possible that an associated delivery factor is encoded elsewhere in the genome, a phenomenon that has been observed for other AB-type toxins. It is also possible that these RIPs have an intrinsic capacity to drive their own uptake into target cells. Interestingly, the RIP encoded by *Streptomyces scabiei* has a C-terminal KDEL sequence, the ER retention/retrieval sequence for animals and for some plant proteins (HDEL is more common in fungi and many plants, but KDEL is often functional in these lineages as well). Although this would not facilitate cellular uptake, this strongly suggests a trafficking pathway for the toxin if it is endocytosed, since C-terminal KDEL sequences are employed by AB-type toxins such as cholera toxin and pertussis toxin to facilitate their trafficking to the cytoplasm via retrograde transport to the ER. The C-terminal KDEL sequence in this RIP strongly suggests that it targets a eukaryotic host. *S. scabiei* is a relatively rare example of a *Streptomyces* phytopathogen, and it is possible this toxin targets a plant host as a virulence mechanism.

### Multi-domain bacterial RIP proteins

**Fig. 3.**
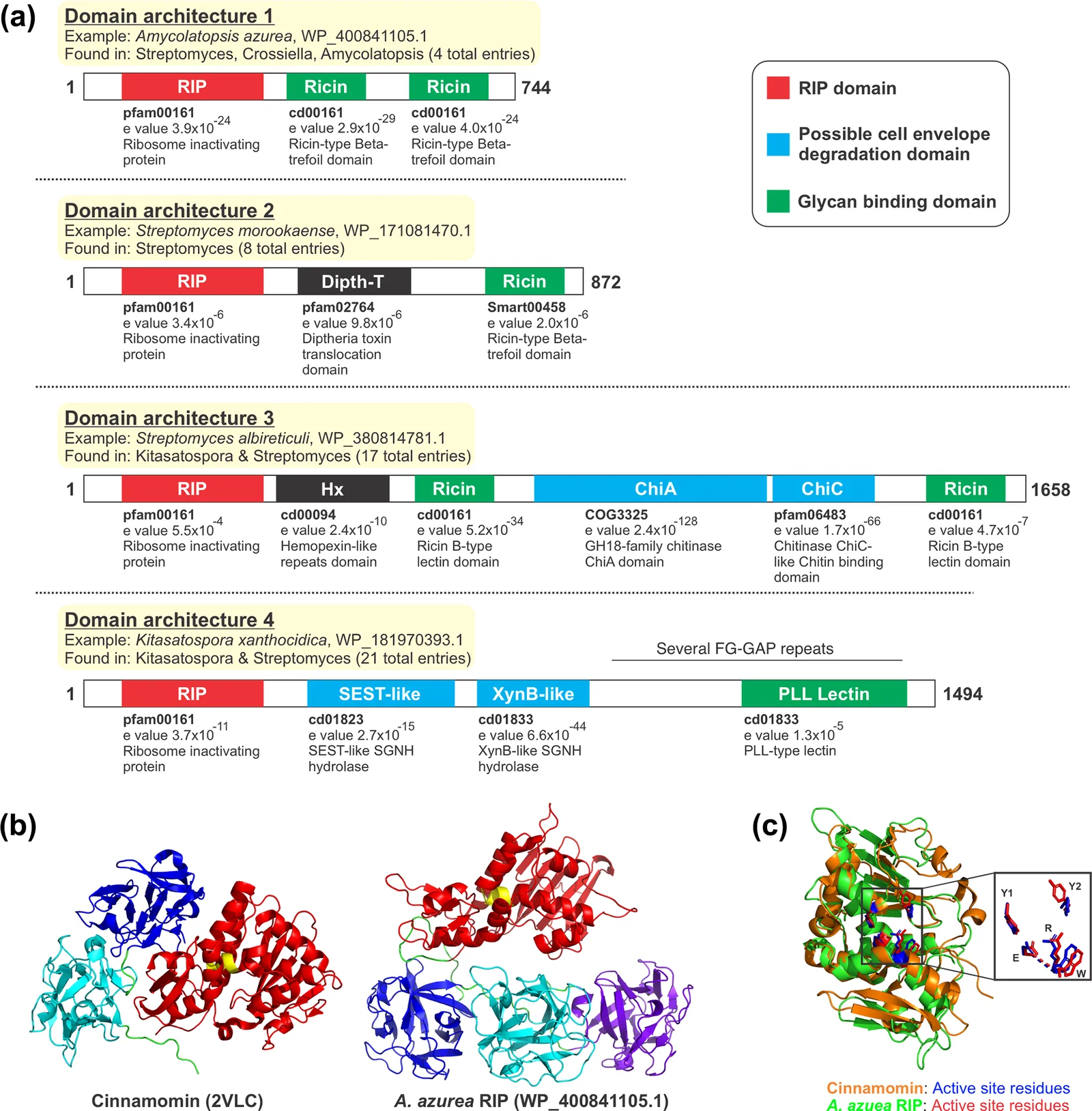
Putative multi-domain RIPs encoded by bacteria. (**a**) The NCBI CDART tool (https://www.ncbi.nlm.nih.gov/Structure/lexington/lexington.cgi) was queried for domain architectures of proteins carrying a RIP domain (pfam00161) within the Bacteria domain, and selected architectures of special interest are shown. Protein diagrams show coloured segments to represent the identified conserved functional domains, colour-coded according to the legend, where black domains do not fit into any of the assigned categories. Beneath each conserved domain, the domain identifier is provided along with e values informing on the confidence that the domain is present in the queried protein and a brief descriptive name for the domain. (**b**) Ribbon diagrams showing the experimentally determined structure of the type 2 eukaryotic RIP cinnamomin (PDB entry 2VLC) and the predicted structure of WP_400841105.1, a putative RIP encoded by *A. azurea* strain NPDC090503 highlighted in (**a**). Structural prediction for WP_400841105.1 generated using AlphaFold 3 [152]. Putative domain architecture shown by colour-coding, where the blue and purple domains represent ricin-like lectin domains and the RIP domain is shown in red, with intervening loops shown in green. The short segment containing the RIP catalytic motif (ExxR) is shown in yellow. (**c**) Alignment of the two structures from (**b**), performed using the PyMOL Molecular Graphics System (Version 2.5.2, Schrödinger LLC, https://pymol.org), showing the structurally similar catalytic cores of the RIP domains. The inset shows the locations and orientations of the five conserved RIP active site residues discussed in the main text and in Fig. 2(b).

To explore the possibility that, similar to type 2 RIPs from plants, certain bacterial RIP domains are found as part of larger proteins that also include a delivery mechanism, we used the NCBI Conserved Domain Architecture Retrieval Tool (CDART) [143]. For a given conserved protein domain (the RIP domain in this case), this tool identifies protein architectures – combinations of conserved protein domains – that are found in proteins from the NCBI sequence database. CDART revealed numerous domain architectures for bacterial RIPs. Notably, this includes a domain architecture that matches that of type 2 RIP proteins (Fig. 3a), featuring a RIP domain and ricin-like lectin domains. This architecture was only observed in four bacterial proteins, but these proteins are found in three different genera of *Actinomycetes* (*Streptomyces*, *Crossiella* and *Amycolatopsis*). We further analysed the *Amycolatopsis azurea* representative to determine how it compares to eukaryotic type 2 RIPs. blastp revealed that this protein shares limited sequence similarity to any eukaryotic protein (top hits shared ~30% identity over ~40% query coverage), suggesting that this putative RIP is evolutionarily distant from all known eukaryotic RIPs. However, an AlphaFold-generated structure of the *A. azurea* RIP showed a similar global structure to the experimentally determined structure of the type 2 eukaryotic RIP, cinnamomin [144] (Fig. 3b). The catalytic core of the RIP domains for these two proteins align very closely, with the key active site residues predicted to be arranged in almost identical orientations (Fig. 3c). The lectin domains exhibit similar overall structures, although they are arranged differently compared to the RIP domain in this predicted structure, and the *A. azurea* protein has a third putative lectin domain (Fig. 3b). This suggests *Actinomycetes* encode proteins that are structurally and architecturally similar to eukaryotic type 2 RIPs. Interestingly, this *A. azurea* RIP exhibits only ~30% aa sequence identity with the *Streptomyces* RIP identified as having the same ricin-like domain architecture, suggesting that bacteria produce diverse proteins with a classic type 2 RIP architecture previously thought to only exist in Eukarya.

In addition to architectures similar to type 2 RIPs, other bacterial RIPs with interesting architectures were identified, a sampling of which is shown in Fig. 3(a). This includes an architecture that features a RIP domain and a ricin-like lectin domain flanking a central diphtheria toxin translocation domain. In diphtheria toxin, following receptor binding and endocytic uptake, this domain inserts into the membrane of the acidified endosome, forming a pore through which the associated toxin can escape [145]. This suggests a mechanism wherein this putative toxin triggers its endocytosis into target cells using its lectin domain and subsequently uses its central translocation domain to enable the RIP to escape the endosome and gain access to the cytosol. Interestingly, certain *Streptomyces* were recently shown to produce diphtheria-like toxins, which were proposed to target insects as a virulence mechanism [146]. Two other architectures highlighted in Fig. 3(a) are large proteins wherein RIP and lectin domains are combined with putative cell envelope degrading enzymes such as chitinases or SGNH hydrolases that are similar to enzymes that degrade glycans or lipids. Coupling domains similar to type 2 RIPs with degradative enzymes could be a way to combine two independent toxic activities into a single secreted toxin, or alternatively, the degradative enzymes could potentially clear away cell envelope components that would otherwise obstruct access of the toxin to its receptors.

### The evolutionary origins of RIPs

It was historically assumed that the RIP functional domain evolved within plants. More recent models have called this premise into question, as the expansion of DNA sequence databases has shown that RIPs are spread across the tree of life [128]. Many bioinformatic approaches commonly used to infer phylogenetic relationships (e.g. phylogenetic trees) are not effective when it comes to mapping the origins of RIPs. From a sequence similarity perspective, the most divergent RIPs have likely diversified to the maximum extent that is possible while still retaining RIP activity. This is exemplified by our analysis here (Fig. 2) where ten divergent RIPs all share ~20% aa sequence identity, and much of this identity maps to residues important for the core structure or activity of the enzyme. The minor differences in sequence similarity when comparing divergent RIP sequences are more likely to be ‘noise’ than evidence of *bona fide* evolutionary relationships.

We propose a model wherein the RIP domain evolved within an ancient ancestor of modern *Actinomycetes* as a defence or competition mechanism against (most likely eukaryotic) microbes. This model is speculative but is supported by several observations. This includes *Actinomycetes*’ unmatched pedigree for evolving to produce novel toxic molecules and, most importantly, the extraordinary levels of RIP diversity in contemporary *Actinomycetes*, particularly *Streptomyces*, as illustrated above. Furthermore, *Actinomycetes* have many advantages compared to plants when it comes to *de novo* evolution of novel toxic proteins, including their extremely large numbers, their short generation time and their propensity for genetic flexibility. Regardless of where RIPs first evolved, *Streptomyces* have presumably been a central player in the spread of this functional domain throughout the biosphere. The ecological distribution of *Actinomycetes* (*Streptomyces*) would provide an opportunity to spread RIPs to many of the lineages where they are present today. Organisms that produce RIPs (e.g. plants and fungi) predominantly live in the soil where *Streptomyces* are ubiquitous. *E. coli* and *Shigella* are not endemic to the soil and aquatic environments where *Streptomyces* are most prevalent; however, these do represent important niches occupied in the short term as they are transmitted from host to host, providing the opportunity for genetic exchange. *Streptomyces* are also commonly present in insect microbiomes [147], providing opportunities for genetic exchange with *Spiroplasma*. The presence of both type 1 and type 2 RIPs in *Actinomycetes* suggests the possibility that both RIP types may have independently been transferred into plants. Indeed, the R-type lectin domain found in type 2 RIPs can also be found in bacterial carbohydrate-degrading enzymes, including well-characterized examples from *Streptomyces* [148], which is a logical possibility as the evolutionary origin of this delivery motif.

How might Shiga toxin have evolved? Our preferred hypothesis is that a RIP-containing toxin that originated from *Actinomycetes* (e.g. *Streptomyces*) was transferred, directly or indirectly, into *E. coli* or an associated phage. There are many possibilities for how a *Streptomyces* RIP could have evolved into the Shiga toxin that exists today. It is possible that the ancestral RIP was an AB_5_-type toxin, although we do not prefer this hypothesis given the lack of known AB_5_-type toxins in *Streptomyce*s and the abundance of such toxins in *E. coli*. The hypothetical ancestral RIP could have been incorporated into an AB_5_ toxin and transferred into *E. coli* via a complex multiple-step process, and/or involving species whose genomes have never been sequenced, or which have gone extinct. However, a straightforward model for how this might have occurred would be that a *Streptomyces* RIP was transferred into *E. coli* and subsequently evolved to form a complex with the delivery subunits from an existing AB_5_ toxin, supplanting the existing A subunit and forging the precursor of contemporary Shiga-family toxins. This possibility is supported by what is known about AB_5_-type toxin distribution and evolution. *E. coli* is home to the largest and most diverse assortment of known AB_5_-type toxins of any bacterial lineage [48]. In addition to Shiga toxin, this includes the (cholera toxin-like) heat-labile toxin, simple pertussis-like toxins and subtilase toxin. Notably, subtilase toxin appears to have evolved as the result of a novel enzyme supplanting the A subunit of an existing AB_5_-type toxin [149], and similar evolutionary events yielding new AB_5_ complexes have been proposed for *Salmonella* typhoid toxins [150, 151]. This provides a precedent for this proposed model, although it is worth noting that other AB_5_-type toxins with Shiga toxin-like B subunits have not yet been identified, so the evolutionary source of StxB remains a mystery.

### Concluding remarks

RIPs are celebrated plant toxins, but our appreciation for RIPs in bacteria was long limited to Shiga toxin, which appeared to be an anomaly. The more recent discovery and characterization of *Spiroplasma* RIPs as factors that promote their endosymbiotic relationship with their host have provided a second (and very different) example of how bacteria have evolved to exploit the potent toxicity of RIPs. In both of these examples, RIPs have a profound impact that spreads beyond the bacteria that produce them. Shiga toxin has a substantial impact on human health, while *Spiroplasma* RIPs are proposed to have significant ecological impacts if their activities disrupt the capacity of parasitic species to prey on their hosts. With the wealth of genomic sequencing data that is now available, it is evident that, although they are relatively rare across the Bacteria domain, RIPs are broadly distributed across diverse bacterial taxa and are common in certain lineages. Some such RIPs, analogous to Shiga toxin, might represent important toxins for bacterial pathogens of other animals. For example, *A. salmonicida* is a globally important fish pathogen that strongly impacts salmonid farming and conservation. Given the potent toxicity of RIPs, it would be interesting to assess the role of the putative RIPs encoded by this species in their capacity to cause disease. Other RIPs could potentially be produced by bacteria to target other microbes that are predators or competitors. This is our preferred hypothesis for the role of the considerable collection of RIPs encoded by diverse *Streptomyces* strains. If this hypothesis is correct, research that sheds further light on the activity and function of *Streptomyces* RIPs could unveil novel mechanisms through which *Streptomyces* alters community dynamics using secreted protein toxins rather than the small molecule antibiotics that are more commonly considered to be their predominant antimicrobial weapons.

Beyond their natural function, the potent toxicity of RIPs makes them attractive candidates for a range of applications. RIPs are a concern as potential agents that could be used in biological warfare or bioterrorism [46]. There are rare but well-publicized cases of ricin being used nefariously, such as a letter laced with ricin that was sent to then-US President Barack Obama in 2013, and ricin being used to assassinate Bulgarian dissident Georgi Markov in 1978. In contrast to these sinister uses, RIPs have strong potential for a range of beneficial applications in agriculture or medicine [1, 47]. For example, there has been substantial interest in fusing RIPs to proteins that mediate their specific delivery to malignant cells (e.g. monoclonal antibodies) as a cancer treatment. Although the resulting therapeutics have thus far been stymied by a variety of issues ranging from production issues to immunogenicity, this approach has shown significant promise as well. The large collection of uncharacterized bacterial RIPs represents an untapped resource for future RIP-based applications. For example, a RIP with an efficient delivery mechanism and which exhibits specificity for fungal organisms would be a promising tool for the development of novel antifungal technologies.

## Abbreviations

CDART: Conserved Domain Architecture Retrieval Tool
EHEC: enterohaemorrhagic *Escherichia coli*
HUS: haemolytic uraemic syndrome
LEE: locus of enterocyte effacement
PDB: Protein Data Bank
RIP: ribosome-inactivating protein
RSR: ribotoxic stress response
STEC: Shiga toxin-producing *Escherichia coli*
